# Influence of technique used to attach the infusion set to peristaltic finger smart-pumps on dispensing time: an experimental study

**DOI:** 10.1186/s40780-018-0104-4

**Published:** 2018-04-16

**Authors:** Masayuki Umemura, Kanae Maegawa, Daichi Arai, Katsuro Shigeno, Yoshifumi Wakiya

**Affiliations:** 1 Laboratory of Pharmacy Practice and Sciences, School of Pharmacy, Aichi Gakuin University, 1-100 Kusumoto-cho, Chikusa-ku, Nagoya, 464-8650 Japan; 2 Department of Pharmacy, Tajimi Municipal Hospital, 3-43, Maehata-cho, Tajimi, 464-8650 Japan

**Keywords:** Peristaltic finger, Infusion pump, Smart pump, Polyvinyl chloride infusion set, Polybutadiene infusion set, Stretching, Traction load, Dispensing rate, Attaching procedure

## Abstract

**Background:**

Infusion sets designed for peristaltic finger smart pumps (PFSPs) are necessary for the pumps’ accurate handling. We previously found that medication dispensing is occasionally incomplete following the calculated infusion time when using certain combinations of PFSPs and infusion sets at a Japanese hospital. Thus, in this study, we investigated the cause of this observed delay by determining the effect of infusion set attachment technique on dispensing time using a combination of three kinds of PFSPs and five kinds of polyvinyl chloride (PVC) and polybutadiene (PB) infusion sets.

**Methods:**

PFSPs with their exclusive infusion sets were used. The PVC and PB infusion sets were either not stretched or stretched to 1–3 cm and attached to the PFSP’s liquid delivery system. PFSP dispensing rates were set at 25–400 mL/h. The primary outcome was the time required to dispense 100 mL of saline in a volumetric flask.

**Results:**

The complete dispensing time correlated with the input time for all equipment combinations when the infusion sets were not stretched before attachment to the PFSP (R^2^ = 0.9998–1.0000). When stretched, the complete dispensing time was longer than the input time (*P* < 0.01–0.05, analysis of variance with Tukey-Kramer multiple comparisons). The maximum dispensing time extension ratio for the PVC and PB infusion sets was 141.8% and 113.0%, respectively.

**Conclusion:**

Certain attachment techniques for infusion sets can adversely prolong drug dispensing time. As such, pharmacists should provide medical staff with information about the devices used to administer drugs, as well as about the drugs themselves.

## Background

Infusion pumps are used to strictly manage the dispensing rate of medicine, and thereby improve medical care safety [1–3]. However, a number of factors can influence the accuracy of continuous intravenous dispensing, including the characteristics of the pharmaceutical product and its additives, medical staff’s affinity with medical equipment and the infusion set in particular, staff’s actual handling of the medical equipment, and the conditions in which staff must use this equipment [4–6]. For instance, previous studies have highlighted problems caused by interactions between certain pharmaceutical products and certain infusion pumps in Japan [7–10]. Several Japanese studies have shown that delays in dispensing can result from using pharmaceuticals containing surfactants and a dropping-control-type infusion pump [8–11]. However, no dispensing delay was found when utilizing peristaltic finger smart-pumps (PFSPs) with surfactants [10]. PFSP is a type of infusion pump that relies on finger presses to administer the solution. When a button is pressed, the flow control system inside the tubing attached to the PFSP pushes the solution through the tubing in peristaltic waves [10]. Thus, the inside and outside diameters of the tubing of the infusion set are related to the dispensing rate [12].

In most cases in Japan, the infusion sets for PFSPs and the PFSPs themselves are designed by the same manufacturer. Occasionally, however, medical staff cannot choose a PFSP for a specific infusion set because the manufacturer of the infusion set does not produce a PFSP, or because hospital policy dictates the exclusive use of certain infusion sets for PFSPs, regardless of the PFSPs’ manufacturer. Previously, we reported that there were delays in the time required for complete dispensing when polyvinyl chloride (PVC) infusion sets made by Covidien were attached to PFSPs made by Terumo Corporation [12]. We suggested that the cause of these delays is likely the medical staff’s technique in attaching the infusion set to the Terumo-made PFSP, rather than the interaction of the drugs and its additives. Accordingly, we should verify this finding using multiple PFSPs and infusion sets. Aside from this, there appear to be no reports on the reasons for the delay in dispensing time when using a combination of PFSPs and PVC infusion sets, or whether a delay occurs for frequently used polybutadiene (PB) infusion sets. Therefore, in this study, we investigated the cause of the delay in the time required for complete dispensing by focusing on the effect of the attaching procedure under various conditions, using a combination of three kinds of PFSPs and five kinds of PVC and PB infusion sets.

## Methods

### Medical equipment and chemicals

The PFSPs were manufactured by Terumo Corporation (model TE-161S), Nipuro Corporation (model FP-1200s), and JMS Corporation (model OT-888). The infusion sets made exclusively for use with these PFSPs were used, including the Safe Access infusion set (Covidien Japan), Shuaplug infusion set (Terumo Corporation), JMS infusion set (JMS Corporation), TI infusion set (Toray), and Nipuro infusion set (Nipuro Corporation); each of these products has PVC and PB versions. The saline used was Terumo Seishoku 500 mL (Terumo Corporation). As it was not our intention to evaluate the performance of the specific PFSPs with each infusion set, we have referred to the PFSPs and infusion sets as PFSPs I–III and infusion sets A–E, respectively, in the figures and tables. The inside and outside diameters of these infusion sets are shown in Table 1.

**Table 1:** Inside and outside diameters of infusion set tubing for the peristaltic finger smart pumps

Infusion set manufacturer	Diameter	Diameter after the traction amount to 3 cm
	Inside diameter (mm) × Outside diameter (mm)	
Company A	3.20 × 4.50	2.95 × 4.15
Company B	3.15 × 4.50	2.87 × 4.10
Company C	3.10 × 4.50	2.82 × 4.10
Company D	3.00 × 4.50	2.73 × 4.10
Company E	3.10 × 4.50	2.85 × 4.15

### Effect of interaction between infusion set and PFSP dispensing rate on dispensing time

We set up five of each of the three PFSPs with the attached infusion sets (*n* = 5) for use in this experiment. Both the PVC and PB types of infusion set were used. The PFSPs were attached about 100 cm from the bottom of a 180-cm drip stand, and the infusion sets were attached to the PFSPs according to the instruction manual after setting the dispensing rate to 25, 50, 100, 200, or 400 mL/h. The dispensing time was measured as the time to fill 100 mL of saline in a volumetric flask; for each combination, this time was measured 5 times via observation. The room temperature was maintained at 28 ± 1 °C.

### Effect of the interaction between infusion set attachment method and PFSP dispensing rate on dispensing time

#### Experimental set-up of stretching and attaching the infusion set to the PFSP

**Fig. 1:**
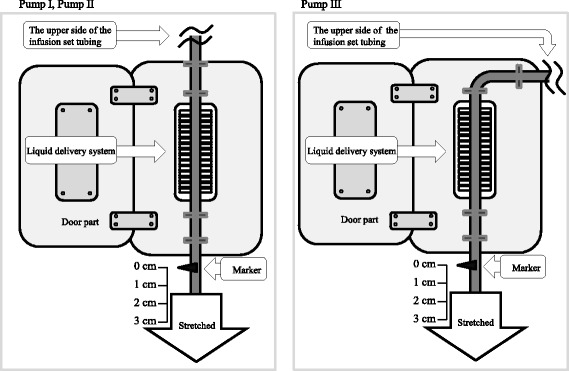
Method of stretching and attaching the infusion set to the peristaltic finger smart pump. The upper part of the infusion set is fixed 1 cm from the pump. The lower part is stretched and attached to the liquid delivery part of the pump at markers of 0, 1, 2, and 3 cm (the traction amounts). After choosing the traction amount, the dispensing rate is set [16]

The instruction manuals of PFSPs I–III mention only to “attach [the infusion set to the PFSP] straight by pulling softly” and “ensure it is not too loose and do not pull too hard.” The total length of the tubing of each infusion set was 30 cm from the liquid delivery system. While the upper part of the infusion set was fixed at 1 cm from the PFSP, the lower part was pulled and attached to the liquid delivery system at markers of 0, 1, 2, and 3 cm (indicating the amount of traction). After attaching the infusion set at the chosen amount of traction, the dispensing rate was set at 25–400 mL/h (Fig. 1).

#### Interaction between amount of traction and dispensing rate

The PVC and PB infusion sets were stretched and attached to the liquid delivery system of the PFSP in the manner described above. We calculated the average dispensing extension ratio (see below for calculation) for infusion sets A, B, C, D, and E for dispensing rates of 25, 50, 100, 200, and 400 mL/h. We then investigated how time extension ratio differed according to traction amount (0–3 cm).

#### Influence of dispensing rate on time extension ratio

For this analysis, we used both the PVC and PB infusion sets for all three PFSPs, set up as in section 1), but constrained the traction amount to 3 cm. Only the dispensing rate varied here (50, 100, and 200 mL/h). We then compared the time extension ratio between the three dispensing rates.

### Statistical analyses

We calculated the mean (SD) time extension ratios for both types of infusion set (PVC and PB). The time extension ratio (%) was calculated as the time required for complete dispensing divided by the input time (i.e., the set dispensing rate for the PFSPs). A time extension ratio of 100% indicated that the time required for complete dispensing was the same as the input time. Statistical differences between conditions were calculated with the paired t-test and analysis of variance (ANOVA), with multiple comparisons using the Tukey-Kramer method. A *P*-value of < 0.05 was considered significant. Data entry and analysis were performed using IBM SPSS Statistics 19 (IBM Japan Ltd., Tokyo, Japan).

## Results

### Interaction between type of infusion set and dispensing rate

**Fig. 2:**
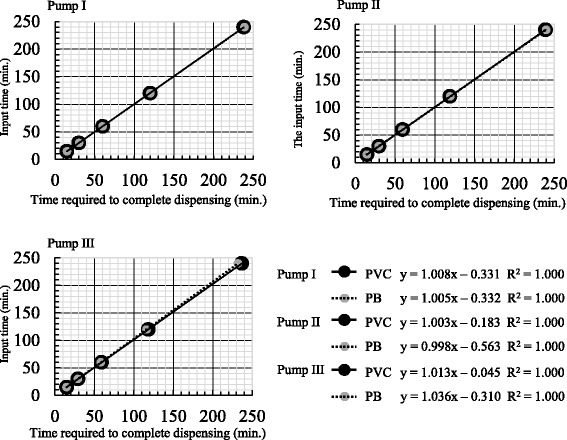
Influence of infusion set type on dispensing rate accuracy for the peristaltic finger smart pumps. The infusion sets are attached according to the pump manufacturer’s instructions. Then, the time required to dispense 100 mL, at dispensing rates of 25–400 mL/h, is measured (mean ± SD, *n* = 5)

We first determined the accuracy of the three PFSPs when they were attached to each of the five PVC and PB infusion sets, using dispensing rates of 25, 50, 100, 200, and 400 mL/h (Fig. 2). The mean ± SD of the accuracies of the PVC and PB sets ranged from 98.7% ± 1.3 to 100.2% ± 2.2 and 96.6% ± 2.4 to 100.7% ± 3.4, respectively. Furthermore, for all combinations of infusion set and PFSPs, the input time and time required for completion were very strongly correlated, such that the regression lines were straight (i.e., R^2^ = 1.000). There were no significant differences in accuracy according to dispensing rate, for either the PVC or the PB infusion sets. Thus, the dispensing rate was accurate for all combinations of PFSP and infusion set.

### Interaction between method of attaching the infusion set and dispensing rate

#### Interaction between amount of traction and dispensing rate

**Fig. 3:**
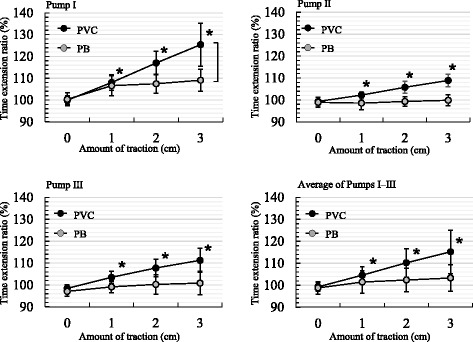
Interaction between traction amounts and dispensing rates for the peristaltic finger smart pumps. The polyvinyl chloride (PVC) and polybutadiene (PB) infusion sets are stretched (0–3 cm) and attached to the infusion pump. Then, the dispensing time is measured at a dispensing rate of 100 mL/h. The time required to completely dispense 100 mL of solution, for all combinations of infusion sets and pumps, was significantly greater than the input time (*P* < 0.01, analysis of variance; mean ± SD, n = 5). The PVC and PB infusion set results were compared using a paired t-test (*P < 0.01). The results of Tukey-Kramer multiple comparisons are shown in Table 2

The results indicated that the PVC infusion sets (for all three PFSPs) showed significantly longer time extension ratios for each dispensing rate as the amount of traction increased (*P* < 0.01, ANOVA) (Fig. 3, Table 2). The time extension ratio of the PVC infusion sets (for all three PFSPs) was highest, at 141.8%, for a dispensing rate of 100 mL/h and a traction of 3 cm. The PB infusion sets also showed significantly greater time extension ratios as the amount of traction increased for each dispensing rate (*P* < 0.05, ANOVA). The time extension ratio was highest at 113.0% for a dispensing rate of 100 mL/h and a traction of 3 cm. This was smaller than that for the PVC. The average time required for complete dispensing of 100 mL was 68.9 and 59.9 min for the PVC and PB infusion sets, respectively, while the time extension ratios were 115% and 103%. We also observed significant differences in the time extension ratio between the PVC and PB infusion sets at all dispensing rates and traction amounts. Specifically, the ratios were consistently smaller for the PB sets than for the PVC sets. Consequently, the dispensing time for PVC infusion sets was significantly prolonged compared to that for the PB sets (*P* < 0.0001).

**Table 2:** Tukey-Kramer multiple comparisons of time extension ratios (%) between the pumps, infusion sets, and traction

Traction amount comparison (cm)	Infusion set material	Pump I (mL/h)					Pump II (mL/h)					Pump III (mL/h)				
		25	50	100	200	400	25	50	100	200	400	25	50	100	200	400
0 vs 1	PVC	8.7*	8.1*	8.2 *	10.2*	10.6*	5.5*	5.4*	5.9*	7.4*	4.7*	2.5*	3.0*	2.3*	3.0*	4.1*
0 vs 2	PB	6.5*	6.0*	6.2*	7.3*	7.6*	2.3	1.9	2.0	2.0	1.5	1.0	1.3	0.2	0.4	0.4
	PVC	16.9*	16.3*	16.7*	18.1*	18.5*	9.2*	10.4*	10.2*	11.8*	8.9*	5.9*	6.3*	6.0*	6.3*	8.3*
0 vs 3	PB	6.7*	6.2*	7.3*	7.9*	8.1*	3.2*	2.6**	2.9**	2.5**	2.0	0.6	0.1	0.4	0.1	0.4
	PVC	24.4*	23.8*	25.3	24.4*	24.9*	11.5*	12.4*	13.5*	14.6*	13.9*	10.5*	9.7*	9.3*	10.4*	11.9*
1 vs 2	PB	9.4*	8.9*	9.0*	8.7*	8.9*	4.0*	3.8*	3.4**	2.5	2.0	0.6	0.0	0.8	1.0	1.0
	PVC	8.2*	8.2*	8.5*	7.9*	7.9	3.7*	4.9*	4.4*	4.4*	4.2*	3.3*	3.3*	3.7*	3.3*	4.2*
1 vs 3	PB	0.2	0.2	1.1	0.5	0.5	0.8	0.7	0.9	0.5	0.4	0.4	1.3	0.6	0.3	0.0
	PVC	15.7*	15.7*	17.1*	14.2*	14.2*	6.0*	6.9*	7.6*	7.2*	9.3*	7.9*	6.7*	7.0*	7.4*	7.9*
2 vs 3	PB	2.9**	2.9**	2.7	1.3	1.3	1.6	1.8	1.4	0.5	0.4	0.4	1.2	1.0	1.3	0.6
	PVC	7.5*	7.5*	8.6*	6.4*	6.4	2.3	2.0	3.3**	2.8	5.0*	4.6*	3.4*	3.3*	4.1*	3.7*

The numbers indicate increases from the average time extension ratio (%), which is the average for all infusion sets at each dispensing rate for Pumps I–III (*P < 0.01, **P < 0.05). The differences in time extension ratios are shown in Fig. 3. PVC = polyvinyl chloride, PB = polybutadiene

#### Influence of dispensing rate on time extension ratio

**Fig. 4:**
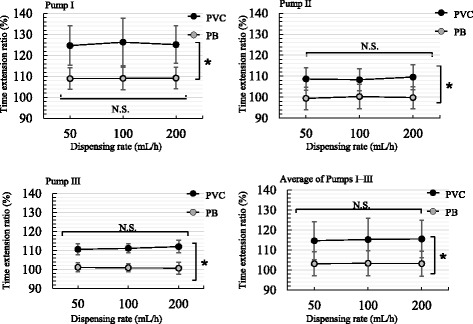
Influence of dispensing rate on time extension ratio for peristaltic finger smart pumps. The polyvinyl chloride (PVC) and polybutadiene (PB) infusion sets are stretched to 3 cm and attached to the infusion pumps; dispensing rates are set at 50–200 mL/h. The times required to completely dispense the solution, for all combinations of infusion sets and pumps, were not significantly longer than the input times (N.S., not significant; analysis of variance; mean ± SD, n = 5). The comparison of PVC to PB infusion sets was performed using a paired t-test (*P < 0.01). The results of the Tukey-Kramer multiple comparisons are shown in Table 3

When constraining the traction to 3 cm, we examined differences in the average time extension ratio at dispensing rates of 50, 100, and 200 mL/h (Fig. 4). Table 3 shows a comparison of the average time extension ratios by dispensing rate for all infusion sets. We found no significant differences in average time extension ratio for the PVC or PB infusion sets between the dispensing rates, according to Tukey-Kramer multiple comparisons. This was true for all five infusion sets (A to E) of each type. However, we did observe significant differences in the time extension ratios between the PVC and PB infusion sets.

**Table 3:** Tukey-Kramer multiple comparisons of the time extension ratios (%) at varying dispensing rates and 3-cm traction

Dispensing rate comparison (mL/h)	Infusion set material	Pump type (%)		
		Pump I	Pump II	Pump III
50 vs 100	PVC	1.9 ± 0.6*	1.5 ± 0.5*	0.6 ± 0.2*
	PB	1.9 ± 0.6*	1.9 ± 0.6*	1.9 ± 0.6*
50 vs 200	PVC	1.4 ± 1.4*	1.3 ± 1.1*	1.0 ± 0.8*
	PB	1.9 ± 0.6*	1.9 ± 0.6*	1.9 ± 0.6*
100 vs 200	PVC	1.4 ± 0.8*	1.4 ± 0.7*	1.1 ± 0.3*
	PB	1.9 ± 0.6*	1.9 ± 0.6*	1.9 ± 0.6*

The numbers indicate increases from the average time extension ratio (%) for all infusion sets. The results of the comparison are shown in Fig. 4. *NS: Not Significant, Mean ± SD

## Discussion

PFSPs have recently begun to be used for accurate continuous intravenous injection in outpatient clinics in Japanese hospitals. However, errors in dispensing rate using PFSPs are continuing to be reported, and often occur because the dispensing rate exceeds the tolerance range of the PFSP [12]. For most PFSPs, the instruction manual indicates that the best results are achieved when the infusion set is designed by the same company as the PFSP. This is because PFSPs rely on peristaltic waves in order to administer the solution; a flow control system inside the tubing attached to the PFSP pushes the solution through the tubing in peristaltic waves upon pressing a button. Thus, the accuracy of the dispensing rate relies on the inside and outside diameters of the infusion set tubing in the liquid delivery system of the PFSP. In this study, we examined the differences in the materials of the infusion set tubing, without considering whether the infusion set was designed by the same company or not. The results showed that the time extension ratio was unrelated to the material of the infusion set and PFSPs—the ratios ranged from 94.7 to 102.3%, with an error margin of about 5%. This suggests that the infusion set does not need to be designed specifically for use with a given PFSP, so long as the inside and outside diameters are appropriate and the infusion set is operating correctly. Therefore, obtaining information on the inside and outside diameters is necessary to use infusion sets made by other companies in clinical practice.

On the other hand, infusion sets can be easily stretched 1 to 3 cm by medical staff. For example, the tension force necessary to extend 30 cm of PVC or PB tubing by about 3 cm is approximately 6.8 Newtons, or 700 g-force. Additionally, we did not find large differences in this tension force among individual infusion sets. We found that the dispensing time was prolonged, regardless of the PFSP and material of the infusion set, when the infusion set was stretched before being attached to the PFSP. Furthermore, the time extension ratio increased linearly with the amount of traction. This is likely because the amount of solution sent through the tube decreases when the inside and outside diameter is reduced through stretching the tubing, leading to an increase in the time required for completion. For example, the inside volume of the tubing decreased by 83–85% when the tubing was stretched to 3 cm, as shown in Table 1. In that case, the theoretical dispensing time extension ratio was prolonged by approximately 17–20% (the actual time extension ratios of PVC were 9.3–25.3%). The delay in dispensing was particularly prolonged in the PVC infusion sets as traction increased. The possible reason for this is that PB is more rubber-like than is PVC, and thus more easily returns to its original position. Thus, the delay in dispensing time might hinge on the tolerance level of the material, particularly when traction is high (see Fig. 4). This suggests that the PB infusion sets were superior to the PVC sets for frequently used dispensing rates when the tube was stretched out. Consequently, we need to inform medical staff of the need to avoid pulling the infusion set too hard when attaching PVC tubing (as compared with PB tubing) in clinical practice.

## Conclusions

In previous research, we demonstrated that surfactants such as polyoxyethylene castor oil and polysorbate 80, which are additives of injected agents, and ethanol, which reduces the viscosity of water-based solutions, do not affect the dispensing rate of PFSPs [10, 12]. However, we did not discuss the difference in performance between each PFSP in that study. In this study, we determined that the time extension ratio of various PFSPs is not determined by a particular infusion set or PFSP, but rather depends on the handling technique of the medical staff using the equipment [13–15]. PVC infusion sets rely heavily on plasticizers such as tris(2-ethylhexyl) trimellitate (TOTM) to maintain their softness. Accordingly, PVC tubing is relatively rigid and inflexible compared with PB tubing, which means it is less able to rebound to its original form after extension. Therefore, for PVC infusion sets, it is important to avoid extending the infusion sets when attaching the tubing to PFSP. Pharmacists should draw the attention of other medical staff to this issue. In other words, pharmacists should not only provide information on the drugs that staff will be administering, but also how to handle the medical devices that they will be using.

## Abbreviations

ANOVA: analysis of variance
PB: polybutadiene
PFSPs: peristaltic finger smart pumps
PVC: polyvinyl chloride
TOTM: tris(2-ethylhexyl) trimellitate
